# Cystoid Macular Edema in Acute Presentation of Central Retinal Artery Occlusion

**DOI:** 10.1155/2012/530128

**Published:** 2012-04-04

**Authors:** Wei Yan Ng, Doric W. K. Wong, Ian Y. S. Yeo, Daphne C. Y. Han

**Affiliations:** Department of Ophthalmology, Singapore National Eye Centre, Singapore 168751

## Abstract

A seventy-six-year-old lady with poor vision of the left eye due to previous retinal detachment presented with acute visual loss of her right eye secondary to central retinal artery occlusion. Clinical examination showed a pale right optic disc, macular edema, and a cherry red spot. Optical coherence tomography done four hours after onset showed right acute cystoid macular edema and diffuse inner retinal thickening. Subsequent treatment with intravenous carbonic anhydrase inhibitor resulted in some visual improvement. Central retinal artery occlusion has been known to produce diffuse intraretinal edema instead of cystoids changes. We would like to discuss a case of acute cystoid macular edema in acute central retinal artery occlusion.

## 1. Introduction

Central retinal artery occlusion is a disastrous ophthalmic emergency which presents with an acute painless visual loss and carries a very poor prognosis. Hayreh demonstrated using histological studies that central retinal artery occlusion results in damage to inner retinal areas, while outer retinal areas are preserved [1]. This in turn leads to intra-cellular retinal edema of the innerretinal layer which differs from cystoid macular edema which is extracellular arising between the inner nuclear and outer plexiform layer [1–3]. Apart from a case of aphakic cystoid macular edema in a patient with previous branch retinal artery occlusion described by Friberg and Landers [4], there have been no literature reports of acute cystoid macular edema in acute central retinal artery occlusion. Our case could indicate that the outer retinal layer is involved as well in central retinal artery occlusion.

## 2. Case Report

**Figure 1:**
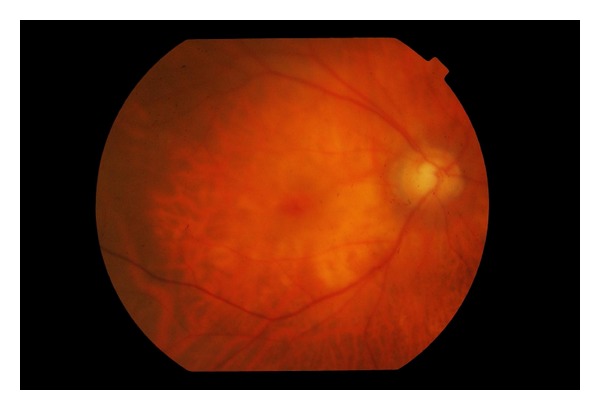
Pale optic disc and macular edema with cherry red spot.

**Figure 2:**
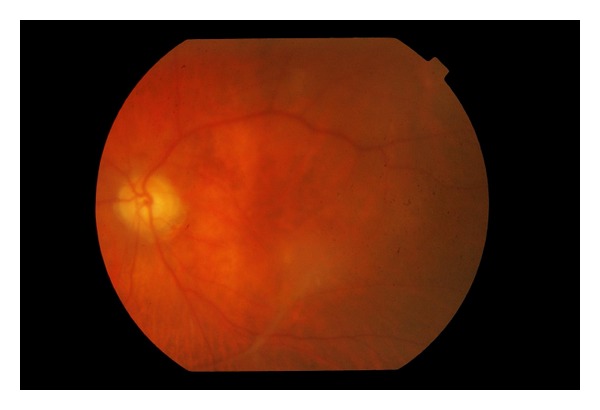
Contralateral fundal photography.

**Figure 3:**
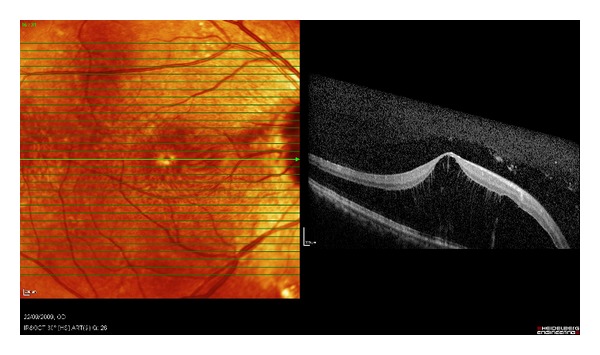
Spectralis OCT showing severe cystoid macular edema with diffuse inner retinal layer thickening in the foveal cut.

**Figure 4:**
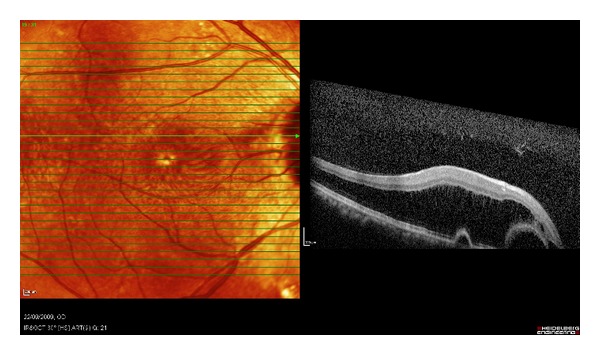
Spectralis OCT showing cystoid macular edema with diffuse inner retinal thickening and two areas of pigment epithelial detachment superior to the fovea.

A seventy-six-year-old lady with a history of hypertension and previous sclera buckle for left eye retinal detachment presented to Singapore National Eye Centre with acute loss of vision of the right eye. There was no prior history of trauma or pain. On examination, visual acuity of the right eye was hand motions in the temporal region and perception of light in all four quadrants with a right grade 2 relative afferent pupillary defect. Intraocular pressure measured by Goldmann Applanation Tonometry was sixteen bilaterally. Anterior segment examination was unremarkable. Fundal examination of the right eye showed a pale optic disc and pale edematous macula with an evolving cherry red spot (Figure 1). There was a very weak retinal venous flow which could be occluded on gentle pressure, indicating that the occlusion was likely incomplete. There were no plaques noted. Fundal examination of her left eye showed an epiretinal membrane and a flat retina with buckle indentation (Figure 2). She underwent immediate ocular massage, anterior chamber paracentesis and intravenous acetazolamide 500 mg was administered. Optical coherence imaging using Heidelberg Spectralis was performed immediately after the above treatments and images showed opacification of inner retina with mild thickening and diffuse edema in the outer retina (Figures 3 and 4). Visual acuity subsequently improved slightly, and she was able to count fingers closely. She was admitted for complete work up to determine the cause of the event, and grave prognosis was conveyed to the patient. Carotid ultrasound showed an immobile 3 mm calcified plaque in the proximal segment of the right internal carotid artery, while 2D echocardiogram did not reveal any vegetations and fasting lipids and glucose were normal. Neurology consult was sought, and she was advised to start on aspirin 100 mg once a day, while no surgical intervention is required for the calcified plaque. Her vision remained stable and did not improve beyond counting fingers during her course of stay. The patient is currently considering left epiretinal membrane surgery for maximization of visual function.

## 3. Discussion

Central retinal artery occlusion is a devastating event and final visual outcome has been generally poor. It has proven to be difficult to manage with poor treatment outcomes. It presents classically as a foveal cherry red spot as a result of diffuse retinal edema in the ganglion cell layer that obscures the underlying choroidal circulation, while the fovea, which is devoid of ganglion cells, allows transmission of the underlying vascular choroidal hue. Histopathological and optical coherence tomography studies have confirmed that the damage occurs in the inner retinal layers resulting in diffuse intraretinal edema of the inner retinal layers [1–3, 5]. Schmidt et al. have further shown that macular thickness resulting from inner retinal edema does not correlate well with eventual functional outcome [6]. However, there have been no reports of acute cystoid macular edema developing in an acute central retinal artery occlusion.

Cystoid macular edema arises due to the breakdown of the blood-retinal barrier causing accumulation of fluid within the retina, specifically between the inner nuclear and outer plexiform layers, resulting in a “flower petal” appearance [7, 8]. The occurrence of cystoid macular edema in our patient suggests that central retinal artery occlusion does not only affect the inner retinal but the outer retinal layer and outer blood-retinal barrier as well. With the experience of our case, it could also be worthwhile to consider optical coherence tomography in the acute stages of central retinal artery occlusion.

In conclusion, our patient illustrates a rare occurrence of cystoid macular edema in acute central retinal artery occlusion on optical coherence tomography. Treatment of the cystoid macular edema could provide some benefits for the patient.
